# Complexity Analysis of Carbon Market Using the Modified Multi-Scale Entropy

**DOI:** 10.3390/e20060434

**Published:** 2018-06-05

**Authors:** Jiuli Yin, Cui Su, Yongfen Zhang, Xinghua Fan

**Affiliations:** 1 Center for Energy Development and Environmental Protection Strategy Research, Jiangsu University, Zhenjiang 212013, China; 2 College of Business, Shanghai University of Finance & Economics, Shanghai 200433, China

**Keywords:** complexity, entropy, carbon market, multi-scale entropy

## Abstract

Carbon markets provide a market-based way to reduce climate pollution. Subject to general market regulations, the major existing emission trading markets present complex characteristics. This paper analyzes the complexity of carbon market by using the multi-scale entropy. Pilot carbon markets in China are taken as the example. Moving average is adopted to extract the scales due to the short length of the data set. Results show a low-level complexity inferring that China’s pilot carbon markets are quite immature in lack of market efficiency. However, the complexity varies in different time scales. China’s carbon markets (except for the Chongqing pilot) are more complex in the short period than in the long term. Furthermore, complexity level in most pilot markets increases as the markets developed, showing an improvement in market efficiency. All these results demonstrate that an effective carbon market is required for the full function of emission trading.

## 1. Introduction

The carbon market is a market in which carbon emission allowances are traded. The price of carbon emission allowances determined by demand and supply in the market is the carbon price. The first carbon emissions trading scheme (ETS) was initiated by the European Union (EU) in 2005. There were 19 ETSs by the end of 2017, which was more than three times of its number in 2012. They covered over 15% of global carbon emissions which account for more than seven billion tons of greenhouse gas emissions equivalent. The coverage would double as China introduced its national carbon trading system in 2018 after more than four years of pilot work [1].

How well the carbon market performs is particularly important for traders, investors as well as policymakers [2]. In a well-performed market, prices at any point in time can “fully reflect“ available information. This is the essence of the Efficient Market Hypothesis (EMH) given by Fama [3]. The EMH has been extensively tested in various markets, such as stock markets, various commodity futures markets, off-the-counter markets, bond markets, options markets [4]. However, the EMH has not been well tested in carbon markets.

Research emphasis on carbon markets has been paid to the EU ETS. Newell et al. [5] sum up the lessons of the carbon market and look ahead to global policy, pointing out that policymaking is essential to the carbon market. Fan et al. [6] study the complexity of the EU carbon market and concludes that the complexity of carbon market corresponds to the extreme socio-political events. Most existing studies confirm the weak efficiency of EU carbon markets. The EU spot carbon market is verified to be fully effective [7]. Yang et al. [8] believe the EU carbon trading market has been characterized by weak-form efficiency. Adopting the variance ratio method, Alberto et al. [9] find the weak efficiency of EUA carbon market in the second stage. However, some authors reject the weak market efficiency of three main exchanges under the EU ETS [10] and the Intercontinental Exchange between 2008 and 2011 [11]. Charles et al. [12] point out that the lack of the cost-of-carry relationship is the reason of inefficiency of main European carbon markets. However, the market efficiency is found to be improved over the period [13]. The EUA future carbon market is found to be not efficient by using event-study methodology [14] but is found to be efficient within one month [15]. Besides the spot and future markets, the efficiency of the EUA options market is also studied [2]. As to the China’s ETS, Lo [16] believes that the implementation of carbon trading in China is of great significance. Zhao et al. [17] show that the market efficiency in China is not satisfactory although the country has made a preliminary achievement in system designs. In a later research, Zhao et al. [18] find signs of restoring market efficiency in four pilot carbon markets.

As known in the literature, there are many types of market efficiency, such as allocative efficiency, operational efficiency, informational efficiency [19]. A market is informational efficient if the current market price instantly and fully reflects all relevant available information. We limit the type of “market efficiency“ to be “informational efficiency“ in this study.

We apply complexity characteristics to measure the efficiency of carbon markets. The reasons lie in the following two aspects. Firstly, complexity characteristic of a nonlinear system not only embraces or is at least closely connected to all other data features [20] but also determines the characteristics of different internal factors and their relationships [21]. Secondly, the carbon market is regarded as a complex system in which traders have different strategic choices and act in complicated ways with mixed, and often intricate incentives [22].

Entropy, together with fractality and chaos, is generally taken as the measurement of complexity [22]. The concept of entropy was originally developed from the classic Shannon entropy [23]. There are various entropy measures such as approximate entropy [24], E-R entropy [25], Kolmogorov–Smirnov entropy [26] and multi-scale entropy(MSE) [27]. MSE outperforms the previous ones in that it considers the multi-scale property of the underlying system, thereby avoiding misguiding results for complexity multi-scale system. It is pointed out that fuzzy entropy provides improved evaluation of signal complexity [28]. However, this study still apply the sample entropy for the continence to compare the complexity between Chinese pilots markets and the EU carbon market [6].

The modified MSE (MMSE) [29] is an improvement of the MSE. The implementation of MSE consists of two steps: (1) Scale extraction and (2) entropy estimation. The coarse-grain procedure [26] is used to determine the scales of data. The sample entropy (SampEn) is generally employed as the entropy measure. It is pointed out that sample entropy estimation presents larger variance for greater scale factor because the coarse-grained time series becomes shorter [30]. The moving average algorithm [29] handles this issue in a good manner for short time series. Using this method, one can estimate the entropy with a better accuracy or get less undefined entropy.

Before the end of 2013, China had already launched seven ETS pilots in five biggest cities of Beijing, Chongqing, Tianjin, Shanghai, and Shenzhen, and two provinces of Hubei and Guangdong [1]. These seven pilots became fully functional before the end of 2015. Considering the relatively short time of the establishment of the market, this paper studies the complexity of carbon market by the modified MSE method. We describe the methods used in Section 2. Then we present the experiment results and discussion in Section 3. The last section provides the overall conclusions.

## 2. Methods

The modified MSE (MMSE) depends on the calculation of the sample entropy in a certain range of scales [29]. The essence of the modified multi-scale entropy method is trying to largely reserve the data length by using a moving-average process. In this process, one generates the new time series by moving a window with a length of the given scale point by point through the entire time series. The system dynamics is now presented by the newly generated time series on different scales. Then the sample entropy algorithm is applied to the generated time series and the MSE is obtained.

### 2.1. Moving-Average

For the return series of carbon price P(i),i=1,2,⋯,N, the moving averaged time series x(i,τ) at the scale factor τ is calculated as

(1) $$x\left(i,\tau \right)=\frac{1}{\tau }\sum_{j=i}^{i+\tau -1}P\left(j\right),\;1\leq i\leq N-\tau +1.$$

### 2.2. Sample Entropy for Each Moving Average Time Series

Sample Entropy [31] is equal to the negative natural logarithm of an estimate of the conditional probability that subseries of length *m* that match pointwise within some tolerance, *r*, will also match when their length is increased by one.

Given the moving averaged sequence {x(i),i=1,⋯,n=N−τ+1} at the scale factor τ (for clarity, we drop the symbol τ in this subsection), we first define a subseries of length *m* as

(2) $$X_{i}=\left\{x\left(i\right),x\left(i+1\right),\ldots ,x\left(i+m\right)\right\}\;i=1,2,\ldots ,n-m.$$

Let the distance $d_{m}\left[X_{i},X_{j}\right]$ between template vectors $X_{i}$ and $X_{j}$ with length *m* be the largest absolute difference between their corresponding elements

(3) $$d_{m}\left[X_{i},X_{j}\right]=\max_{0\leq k\leq m-1}\left|x(i+k)-x(j+k)\right|,1\leq i,j\leq n-m,i\neq j.$$

Then we denote $C^{m}\left(r\right)$ and $C^{m+1}\left(r\right)$ respectively the number of pairs of series of length *m* and m+1 having distance smaller than *r*.

Finally, the sample entropy is calculated by

(4) $$SampEn\left(m,r,N\right)=-\ln\frac{C^{m+1}\left(r\right)}{C^{m}\left(r\right)}.$$

It is clear that SampEn(m,r,N) will be always non-negative. A smaller value of sample entropy indicates less level of complexity or more self-similarity in a data set.

Empirically, the value of entropy is not very dependent on the specific values of *m* and *r* [32]. For small *m*, especially m=2, estimation of SampEn can be achieved with relatively few points [24]. Considering the length of the sample in this study, we select the case m=2 and only this one. The literature uses *r* values between 0.1 and 0.25 of the standard deviation [24,33]. We use r=0.15σ, where σ is the standard deviation of the data points. This study calculates sample entropy values for the scale factors from 1 to 60 (τ=1,5,20,60 represent scale of one day, one week, one month, and one quarter respectively).

## 3. Experimental Results and Discussion

### 3.1. Data

We consider seven pilot carbon markets in China, namely, Beijing, Chongqing, Guangdong, Hubei, Shanghai, Shenzhen, and Tianjin market. These markets were established and functioned sequentially during 2013 and 2014 with the presumed purpose of providing for experiences to its future national scheme. The sample data are daily trading prices obtained from the Carbon Trading Network (http://k.tanjiaoyi.com/), covering the period from the first trading date of each market to the end of the year 2017. Because there are multiple vintage years of carbon allowances in the Shenzhen market, 2014 Shenzhen carbon emission allowances, known as SZA-2014, is selected as the representative carbon price for Shenzhen pilot. The return series $x_{t}=log\left(p_{t}\right)-log\left(p_{t-1}\right)$ is adopted as the experiment data to calculate entropy value, where $p_{t}$ denote the carbon prices on day *t*.

**Figure 1:**
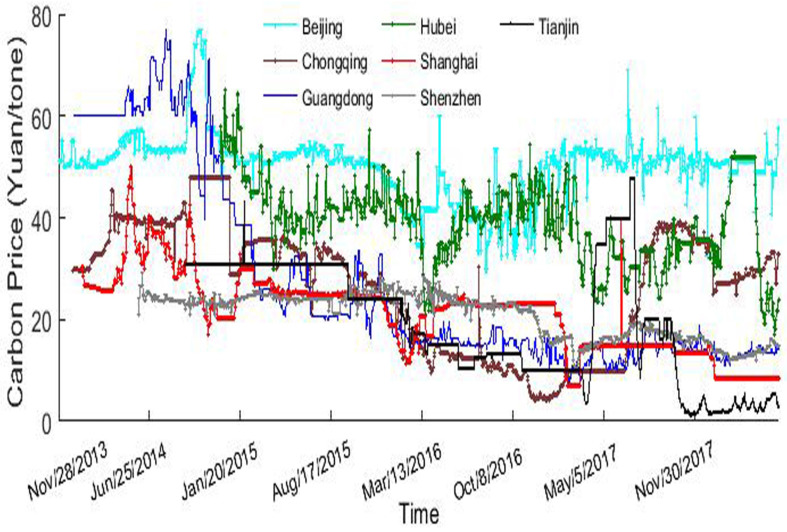
The daily carbon price in seven pilot markets

Figure 1 presents a graphical representation of the sample data. The price in Beijing market is the highest in the most time and fluctuates around 50 Yuan per tonne. The carbon price in Guangdong fluctuates the valiant, ranging from 7.53 to 77 Yuan. Hubei market has a small volatility while markets in Chongqing and Shenzhen have many horizontal segments. The carbon price looks like a radome walk in Beijing, Hubei, and Shenzhen markets while those show a declining trend in Chongqing, Guangdong, and Tianjin markets. All these observations infer that those pilot markets develop at different levels, with Beijing (Hubei and Shenzhen) market be the relatively high-level of complexity while others the low.

### 3.2. Complexity Analysis in Overall Time

**Figure 2:**
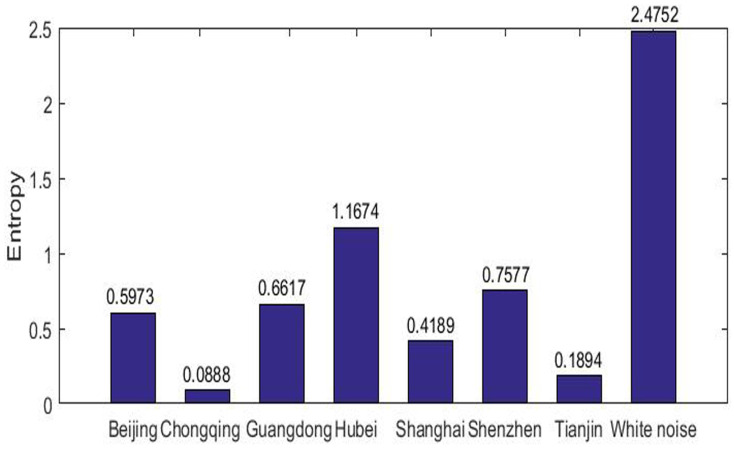
Sample entropy for whole data dynamics.

We consider the complexity from the overall perspective, that is, using the classical sample entropy of the time series without any coarse-grain procedures. For comparison, we consider a white noise with the length to be the largest length of data in the seven pilots. Figure 2 shows the results. Entropy in the pilot markets are all far smaller than that of the white noise (less than a half). Six entropies out of seven are smaller than one with the entropy of Chongqing close to zero and that of Hubei is close to one, meaning that all pilot carbon markets present a low-level complexity. The low-level complexity indicates that all the pilot markets are quite immature in lack of market efficiency. However, marked differences exist among individual markets. It is obvious that Hubei isolates from other markets with the highest entropy of 1.1674, while Chongqing market, accompanied with the market of Tianjin, has the lower entropy around 0.1. We divide the range of the MSE of the pilots equally into three intervals, namely, (0,0.4],(0.4,0.8] and (0.8,1.2] and refer to them as small, medium and large entropy interval respectively. The remaining markets show a medium value of complexity ranging from 0.4–0.8.

### 3.3. Complexity Analysis in Multiple Scales

**Figure 3:**
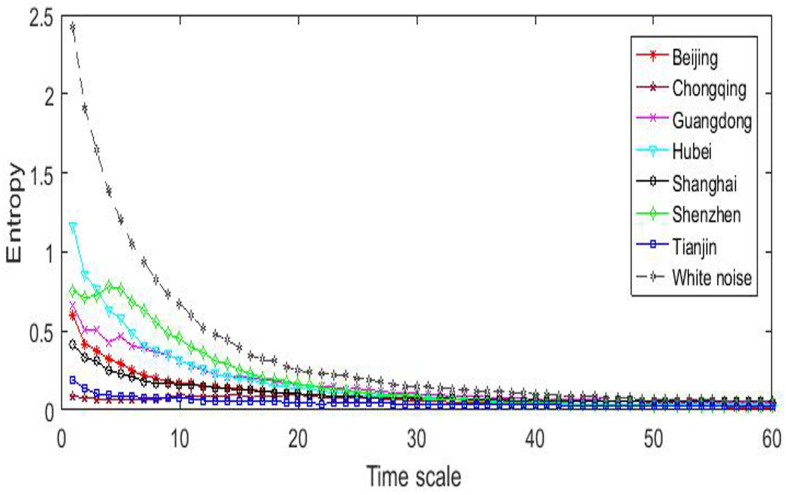
The behavior of multi-scale entropy.

Multiple-scale entropy reflects the complexity from different time scales. Figure 3 reports the results of the multi-scale entropy analysis. Compared with the multiple-scale entropy, the overall perspective is the special case when the time scale is one. This is verified by the coincidence of values in Figure 2 and those in Figure 3 with time scale τ=1.

First, the entropy for every pilot market is monotonically decreasing with the time scale, indicating a decline in complexity level. Second, the curves of the entropy almost level when the time scale is greater than 20. This critical time scale was also found in the European carbon market [6]. These two results suggest that there are different factors affecting the efficiency of the pilot carbon markets. Within the small time scale (shorter than one month) , the inner market features might present more irregular factors leading to the price fluctuations, while fluctuations in a larger time scale (longer than one month) are more related to certain regular (conventional and not occasional) factors or smooth trends.

Third, the rank of complexity changes for some pilot markets. Shenzhen market ranks the first in complexity level for most of short time scales (except for that Hubei ranks the first for the smallest three scales) but is surpassed by Gongdong market for long time scales. Chongqing and Tianjin markets always rank the last for both the short and long time scales. This result suggests that Shenzhen market is the best one in short-term fluctuation while Guangdong market is the best in long-term development, but both Chongqing and Tianjin markets perform the poorest, either in market fluctuation or development.

### 3.4. Evolution of Complexity

**Figure 4:**
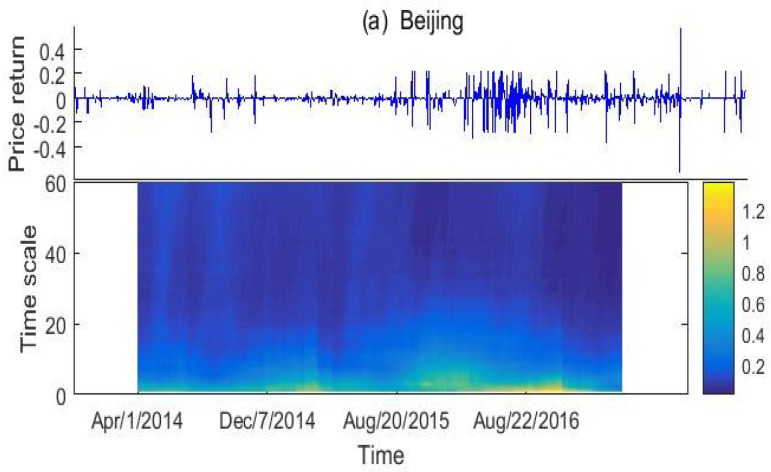

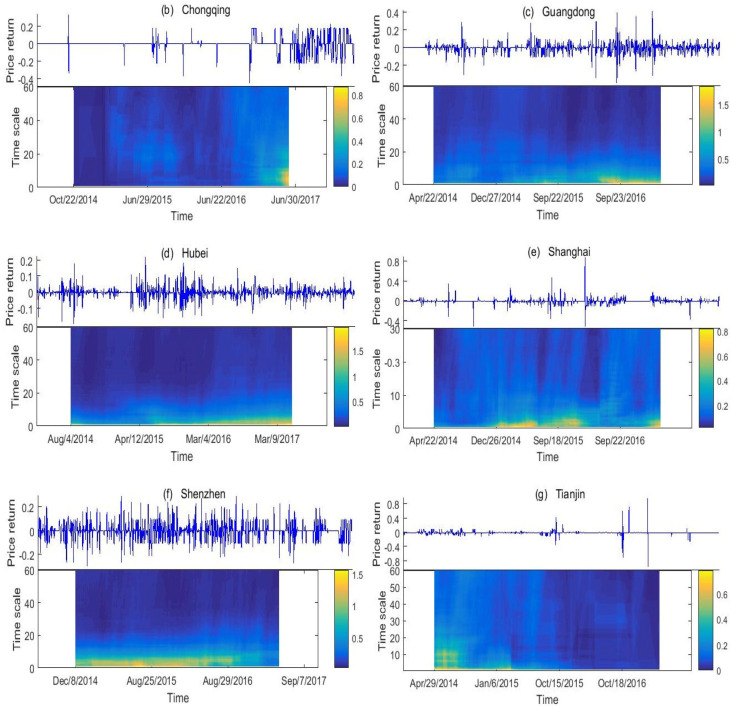
Time dependency of the return series (**upper panel**) and the dependency of the sample entropy to the time and scale (**lower panel**). (**a**) Beijing; (**b**) Chongqing; (**c**) Guangdong; (**d**) Hubei; (**e**) Shanghai; (**f**) Shenzhen; (**g**) Tianjin.

To reflect the dynamics of local situations, rolling window technique is applied to analyze the time-variation of complexity. We use a fixed window width $N_{w}=250$, which is about a year. The step length of the window is set as a single trading day. In detail, we compute the sample entropy of the first window, covering the series from the first data point to the 250th point. Then, the window slides forward by deleting the first point and adding the 251th point. Fixing a scale τ in the interval [2,60], we assign the entropy of the window to its middle point. The results are shown by color diagrams in the lower panel of Figure 4, which also displays the graphes for the corresponding price return series.

There are several common features for the entropy diagrams in Figure 4. First, the diagrams are primarily dominated by large part of small entropies (entropy<0.4). This result confirms that all the markets present a low-level complexity, in lack of mark efficiency. Second, larger entropy locates in the lower part of the diagrams while smaller entropy the upper part. This confirms the finding in Section 3.2 that these markets have a higher complexity in small time scale than in large scale. Third, we note one or three narrow yellow peaks in each diagram. The peak values are higher than 0.8 for Beijing, Guangdong, Hubei, Shenzhen and around 0.7 in other markets. Near those yellow peaks, there are larger regions of other peaks, lower (typically 0.4) than the yellow ones but significantly higher than the other values. Except for Shenzhen and Tianjin markets, those yellow peaks appear at the right end of the time axis, showing global increases in the multi-scale sample entropy with the increase in Chongqing being the most obvious. Those increases indicate that the five markets have improved their market efficiency recently. Fourth, we note a relative large area of yellow peaks for Shenzhen and Hubei markets (Figure 4d,f), covering more than half of the time period at the lower part of the diagrams. This indicates the relatively higher level complexity of the two markets than others. At last, entropy is positively correlated with fluctuation intensity rather than the amplitude of the return series. The more intensive fluctuation, the greater entropy. An apprarent large MSE is observed (Figure 4b) when the return series fluctuates highly after a certain date. Contrast to this, the entropy is close to zero when the return series is mainly flat. Shenzhen and Hubei markets (Figure 4d,f) have much higher entropy than Tianjin (Figure 4g) although amplitudes of their return series are only about one thirds of that in Tianjin.

## 4. Conclusions

We presented a detailed investigation of the correlation of market performance on different time scales. The modified multi-scale entropy by using moving average algorithm was applied to present the complexity of pilot carbon markets in China due to the short length of the sample data.

Our analysis indicates an overall low complexity in those carbon markets, far smaller than that of the Europe carbon market [6]. This inferior market efficiency to that in Europe may be caused by the economic discourse of climate change and excessive state intervention in China’s carbon trading political economy. However, the complexity role was verified in this study by the fact that the complexity of the carbon market (except Chongqing) is higher in small time scales than in large scales. A same critical time scale with the European carbon market was found. Our results also show that complexity is improved as the pilot carbon markets developed while differences in complexity exist.

It is should be noticed that for the time series, the wavelet transform technique [34] may be more efficient in many applications than the moving average technique. One might obtained some different interesting results by using different method.

Results obtained in this study provide a robust basis for investment decisions and policy arrangements. It is also quite important to establish a more consistent quantitative scenario of the recent past.
